# Association between insulin resistance and lung function trajectory over 4 years in South Korea: community-based prospective cohort

**DOI:** 10.1186/s12890-021-01478-7

**Published:** 2021-04-01

**Authors:** Sang Hyuk Kim, Hyun Sam Kim, Hyang Ki Min, Sung Woo Lee

**Affiliations:** 1 Medical Corps 2nd of Armored Brigade, Republic of Korea Army, Paju, Korea; 2 Division of Nephrology, Department of Internal Medicine, Eulji Medical Center, 68, Hangeulbiseok-ro, Nowon-gu, Seoul, 01735 Korea

**Keywords:** Diabetes mellitus, Lung, Insulin resistance, Spirometry

## Abstract

**Background:**

Hyperglycemic conditions are associated with respiratory dysfunction. Although several studies have reported that insulin resistance (IR) is related to decreased lung function, the association between IR and change in lung function has been rarely studied. This study aimed to investigate the potential association of IR on annual change in lung function using a community-based prospective cohort in Korea.

**Methods:**

We selected 4827 Korean participants whose serial lung functions were assessed over 4 years using 1:3 propensity score matching. Exposure was baseline IR estimated with homeostatic model assessment (HOMA-IR), and outcomes were annual changes in lung function determined by calculating the regression coefficient using least-square linear regression analysis.

**Results:**

In the multivariate linear regression, per one unit increased log transformed HOMA-IR was associated with decline in FEV_1_%-predicted (β: − 0.23, 95% CI: − 0.36 to − 0.11) and FVC %-predicted (β: − 0.20, 95% CI: − 0.33 to − 0.08), respectively. In the generalized additive model plot, HOMA-IR showed a negative linear association with annual changes in FEV_1_%-predicted and FVC %-predicted. The suggested threshold of HOMA-IR for decline in lung function was 1.0 unit for annual change in FEV_1_%-predicted and 2.2 unit for annual change in FVC %-predicted. Age showed statistically significant effect modification on the relationship between HOMA-IR and annual change in FEV_1_%-predicted. Increased HOMA-IR was associated with the decreased annual change in FEV_1_%-predicted, particularly in older people.

**Conclusions:**

In South Korea, increased HOMA-IR was associated with decline in lung function. Since IR was related to decline in FEV_1_%-predicted, particularly in older people, tailored approaches are needed in these populations. The potential pulmonary hazard of IR needs to be confirmed in future studies.

**Supplementary Information:**

The online version contains supplementary material available at 10.1186/s12890-021-01478-7.

## Background

Diabetes mellitus is a prevalent chronic disease in modern society, with many experiencing diabetes and its complications [1, 2]. Lung function decline has been considered a major complication of diabetes [3–5]. Several studies have found that diabetes and hyperglycemia are associated with the development of various pulmonary diseases, including chronic obstructive pulmonary disease, asthma, and interstitial lung disease [6–8]. Therefore, good control of diabetes may prevent future lung diseases.

Increased insulin resistance (IR) has been associated with the future development of obesity, diabetes [9], cardiovascular diseases [10], neurologic impairment [11], and kidney dysfunction [12]. Although several studies have reported the potential association between IR and decreased lung function, none of these studies have evaluated the longitudinal effect of IR on future lung function changes. The aging process can cause diverse physiological, immunological and structural changes in the respiratory system [13, 14]. Thus, analyzing consecutive measurements of lung function may help to interpret the effects of aging on IR and lung function changes. In addition, it can clarify a causal association between IR and lung function.

Since diabetes is associated with future decline in lung function and IR is a fundamental pre-condition of diabetes, we hypothesized that increased IR may be associated with future decline in lung function. Therefore, this study aimed to investigate the association between IR and lung function change using data from a community-based prospective Ansan-Ansung cohort in Korea.

## Methods

### Participants

**Fig. 1:**
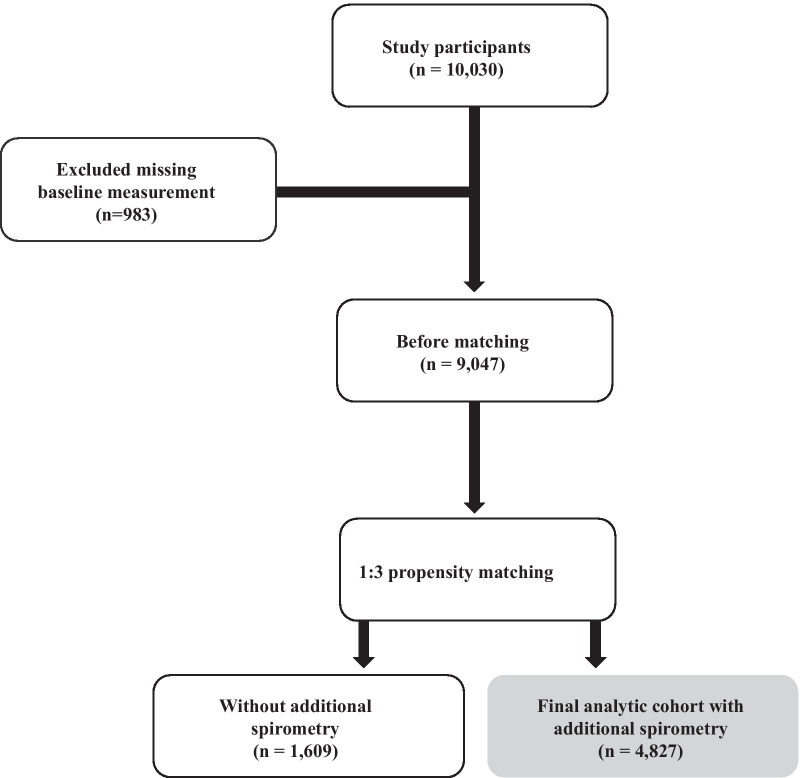
Flow chart of the study subject selection

The Ansan-Ansung cohort comprise participants aged 40–69 years who lived in Ansan (urban) and Ansung (rural) areas, and this cohort was analyzed to determine the factors affecting the incidence of chronic diseases. Baseline measurements were performed between May 2001 and February 2003, and two subsequent lung function tests were performed biennially thereafter. More detailed information about the Ansan-Ansung cohort can be obtained in previous reports [15, 16]. Of the 10,030 participants, we excluded 983 missing baseline measurements. Among the 9047 subjects, 4827 subjects with additional spirometry were selected using 1:3 propensity score matching with 1609 subjects without additional spirometry. The differences in clinical characteristics of participants with or without additional spirometry were described in Additional file 3: Table S1. Therefore, 4827 matched participants were finally included in this analysis (Fig. 1).

### Exposure: IR

IR was assessed using the homeostatic model assessment for IR (HOMA-IR). HOMA-IR was calculated using the following equation: fasting insulin (μIU/mL) × fasting glucose (mg/dL)/405 [17].

### Outcomes: change in lung function

The main outcomes were annual changes in lung function. Lung function tests were performed using a spirometer (VMAX2130; Sensormedics Corporation, Yorba, CA, USA). Pre-bronchodilator values were measured three times by trained technicians, and the best scores were recorded. Predicted values of functional vital capacity (FVC %-predicted) and functional expiratory volume in 1 s (FEV_1_%-predicted) were calculated using the Korean formula [18]. The FEV_1_/FVC ratio was expressed as the percentage of raw value of FEV_1_ divided by the raw value of FVC, according to previous literature [19, 20]. Annual changes in FEV_1_%-predicted, FVC %-predicted, and FEV_1_/FVC ratio was determined by calculating the regression coefficient using least-square linear regression analysis, with FEV_1_%-predicted, FVC %-predicted, and FEV_1_/FVC ratio as functions of time in years. It was applied to all values of FEV_1_%-predicted, FVC %-predicted, and FEV_1_/FVC ratio that was obtained during the follow-up period (Additional file 2: Figure S1).

### Measurements and other definitions

Trained investigators interviewed participants regarding their socioeconomic status and lifestyle habits using Additional file 1: KoGES Baseline Core Questionnaire. High income was defined as the highest quintile of monthly household income (≥ 3 million won a month). Active physical activity was defined as 60 min/day of moderate-intensity activity or 30 min/day of vigorous-intensity activity [21]. Blood pressure (BP) was defined as the average BP on both arms using a standard mercury sphygmomanometer (Baumanometer-Standby; W. A. Baum Co., Inc., Copiague, NY, USA). High BP was defined as systolic BP > 140 mmHg or diastolic BP > 90 mmHg. Body mass index (BMI) was defined by dividing the weight by the square of the height (kg/m^2^). Waist circumference was measured at the narrowest point between the lower rib and the iliac crest (measured to the nearest 0.1 cm). All blood samples were examined after fasting for at least 8 h. Hemoglobin and white blood cell (WBC) count were analyzed using enzymatic methods with ADVIA 120 (Bayer Diagnostics, Tarrytown, NY, USA). Fasting glucose, hemoglobin A1c (HbA1c), triglyceride, high density lipoprotein (HDL) cholesterol, C-reactive protein (CRP), and serum creatinine levels were measured using ADVIA 1650 (Siemens, Tarrytown, NY, USA). Estimated glomerular filtration rate (eGFR) was defined using the Chronic Kidney Disease Epidemiology Collaboration equation [22]. Annual averaged values were obtained for BMI, waist circumference, systolic and diastolic BP, triglyceride, HDL cholesterol, eGFR, WBC count, fasting glucose, HbA1c, hemoglobin, and CRP level.

### Statistical analyses

We compared individuals without additional spirometry or with missing values to the rest of participants. Variables that showed significant differences were selected for matching. As a result, age, sex, high income, college graduate, smoking status, active physical activity, baseline values of BMI, systolic BP, diastolic BP, WBC, CRP, and FEV_1_%-predicted were used in the propensity score matching. The normality of the distribution of continuous variables was assessed using histogram and Q–Q plots. Mean ± standard deviation (SD) was used to express normally distributed continuous variables, whereas median with interquartile range (IQR) was used for non-normally distributed continuous variables. The *P-*trend was analyzed using linear regression for the normally distributed continuous variables, the Jonckheere–Terpstra test for the non-normally distributed continuous variables, and the Cochran–Armitage test for categorical variables. We further analyzed the difference between individuals with and without additional spirometry using student’s t-test for a normally distributed continuous variable, Wilcoxon rank-sum test for non-normally distributed continuous variable, and Pearson’s Chi-squared test for categorical variables. The correlation between age and HOMA-IR was performed using Spearman’s rank correlation analysis.

The simple associations between HOMA-IR and spirometric parameters were expressed using the locally weighted scatterplot smoothing (LOWESS) function. We performed the linear regression analysis between log-transformed HOMA-IR and annual changes in FEV_1_%-predicted and FVC %-predicted. Odds ratio (OR) and 95% confidence interval (CI) for the lowest quartile of annual changes in FEV_1_%-predicted and FVC %-predicted were analyzed using logistic regression analysis.

In multivariate analyses, covariates were chosen based on clinical and statistical relevance. The non-linear association between HOMA-IR and annual changes in FEV_1_%-predicted and FVC %-predicted was analyzed using the multivariate generalized additive model (GAM) for Gaussian distributions with the “mgcv” package, and calculation of Akaike information criterion (AIC) was used for the model fitting. In AIC, lower scores within the data set indicate a better model fit [23]. The threshold point of HOMA-IR was chosen based on the best fit determined by AIC among the models.

In the subgroup analysis, all control variables were used, and participants were divided by the median for continuous variable. The modulatory effect was confirmed by adding the interaction term of variables defining the subgroup to the multivariate linear regression analysis. To visualize potential interaction of age on the association between IR and annual change in lung function, the LOWESS regression with interaction was implemented using the “predict3d” package. A *P*-value < 0.05 was considered statistically significant. All statistical analyses were performed using R version 3.6.2 (R Core Team 2019; R Foundation for Statistical Computing, Vienna, Austria).

## Results

**Fig. 2:**
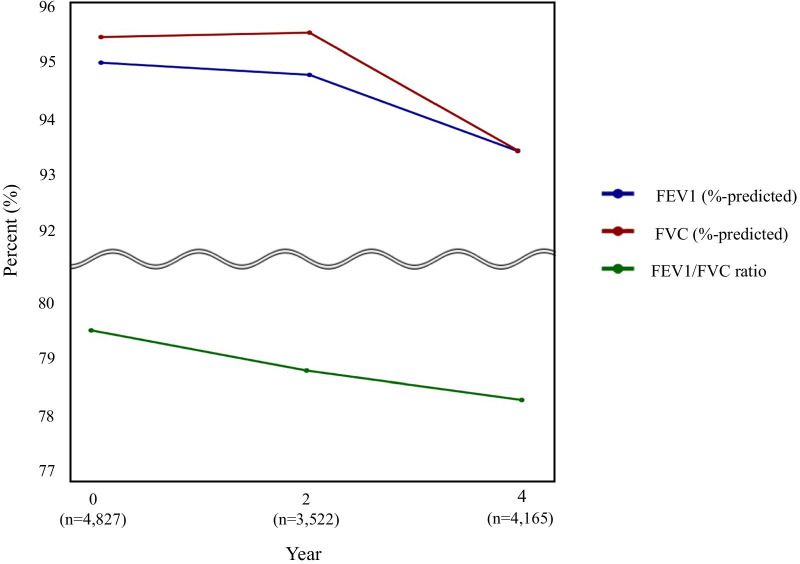
Mean lung function trajectories over 4 years. Abbreviations: FEV_1_, forced expiratory volume in 1 s; FVC, forced vital capacity

The mean age of the 4827 participants was 52.6 years, 45.3% were men, 48.0% had active physical activity, and 41.7% were current or past smokers. The mean ± SD of annual averaged BMI, systolic and diastolic BP, waist circumference, HDL cholesterol, eGFR, hemoglobin, and WBC count were 24.4 ± 3.0 kg/m^2^, 119.6 ± 15.6 mmHg, 79.0 ± 9.4 mmHg, 83.2 ± 8.6 cm, 44.9 ± 8.8 mL/dL, 82.9 ± 10.9 mL/min/1.73m^2^, 13.6 ± 1.4 g/dL, and 6.5 ± 1.5 × 10^3^/μL, respectively. Moreover, the median (IQR) of annual averaged fasting glucose, HbA1c, triglyceride, and CRP level were 88 (83–94) mg/dL, 5.6 (5.3–5.8) %, 130 (97–178) mg/dL, and 0.12 (0.07–0.22) mg/dL, respectively. At baseline, the mean ± SD values of FEV_1_%-predicted, FVC %-predicted, and FEV_1_/FVC ratio were 94.9 ± 14.1% predicted, 95.4 ± 13.2% predicted, and 79.5 ± 7.9%, respectively. The mean of FEV_1_%-predicted, FVC %-predicted, and FEV_1_/FVC ratio decreased in subsequent lung function measurement (Fig. 2). The mean ± SD values of annual changes in FEV_1_%-predicted, FVC %-predicted, and FEV_1_/FVC ratio were − 0.42 ± 2.62%-predicted/year, − 0.49 ± 2.72% predicted/year, and − 0.35 ± 1.56%/year, respectively.

The clinical characteristics of the study population according to HOMA-IR quartile are shown in Table 1. Although age was not associated with HOMA-IR quartile, the increased HOMA-IR quartile was associated with a decreased proportion of men. The correlation analysis between age and HOMA-IR also was not statistically significant (Additional file 2: Fig. S2). Although income and educational status were not associated with HOMA-IR quartile, the increased HOMA-IR quartile was associated with a decreased rate of active physical activity and current or past smoking. With the increase of HOMA-IR quartile, BMI, systolic and diastolic BP, waist circumference, WBC count, fasting glucose, HbA1c, triglyceride, hemoglobin, and CRP level increased, whereas HDL cholesterol decreased. With the increase of HOMA-IR quartile, baseline FVC %-predicted decreased, but FEV_1_/FVC increased. Baseline FEV_1_%-predicted was not associated with HOMA-IR.

**Table 1:** Clinical characteristics of the study population according to HOMA-IR quartile

	HOMA-IR groups (n = 4827)				
	1Q: < 1.05 (n = 1218)	2Q: 1.05–1.45 (n = 1211)	3Q: 1.46–2.03 (n = 1191)	4Q: ≥ 2.04 (n = 1207)	*P*-trend
Age(years)	53.06 ± 9.11	52.53 ± 8.86	52.49 ± 8.84	52.50 ± 8.93	0.131
Male, n (%)	649 (53.3)	551 (45.5)*	462 (38.8)*†	524 (43.4)*‡	< 0.001
High income, n (%)	161 (13.2)	170 (14.0)	160 (13.4)	182 (15.1)	0.263
College graduate, n (%)	119 (9.8)	129 (10.6)	119 (10.0)	127 (10.5)	0.681
Current or past smoker, n (%)	582 (47.8)	494 (40.8)*	433 (36.4)*†	506 (41.9)*‡	< 0.001
Active physical activity, n (%)	595 (48.8)	631 (52.1)*	553 (46.4)†	536 (44.4)*†	0.003
Annual averaged BMI (Kg/m^2^)	23.32 ± 2.95	23.83 ± 2.82*	24.64 ± 2.75*†	25.83 ± 3.03*†‡	< 0.001
Annual averaged systolic BP (mmHg)	117.72 ± 15.32	117.95 ± 15.30	119.49 ± 15.09*	123.44 ± 16.13*†‡	< 0.001
Annual averaged diastolic BP (mmHg)	77.65 ± 9.35	78.05 ± 9.45	79.07 ± 9.17*†	81.14 ± 9.16*†‡	< 0.001
Annual averaged waist circumference (cm)	80.26 ± 8.22	81.63 ± 7.97*	83.78 ± 8.25*†	87.18 ± 8.22*†‡	< 0.001
Annual averaged fasting glucose (mg/dL)	85 (81–90)	86 (82–91)*	88 (83–94)*†	94 (87–109)*†‡	< 0.001
Annual averaged HbA1c (%)	5.4 (5.3–5.7)	5.5 (5.3–5.8)	5.6 (5.3–5.8)*†	5.8 (5.5–6.4)*†‡	< 0.001
Annual averaged triglyceride (mg/dL)	118 (90–162)	120 (91–162)	129 (98–175)*†	154 (115–210)*†‡	< 0.001
Annual averaged HDL cholesterol (mg/dL)	46.47 ± 9.25	45.65 ± 8.91	44.73 ± 8.70*	42.71 ± 8.02*†‡	< 0.001
Annual averaged eGFR (mL/min/1.73m^2^)	83.44 ± 10.98	83.40 ± 10.68	82.43 ± 10.77*†	82.15 ± 11.16*†	0.004
Annual averaged hemoglobin (g/dL)	13.64 ± 1.41	13.51 ± 1.42	13.45 ± 1.51*	13.69 ± 1.42†‡	0.010
Annual averaged WBC count (× 10^3^/μL)	6.40 ± 1.55	6.34 ± 1.47	6.43 ± 1.60	6.78 ± 1.52*†‡	< 0.001
Annual averaged CRP level (mg/dL)	0.10 (0.06–0.20)	0.10 (0.06–0.20)	0.12 (0.07–0.21)*†	0.15(0.08–0.26)*†‡	< 0.001
Baseline FEV1 (L)	2.84 ± 0.68	2.81 ± 0.68	2.77 ± 0.68	2.78 ± 0.68	0.045
Baseline FVC (L)	3.63 ± 0.86	3.57 ± 0.87	3.49 ± 0.86*	3.49 ± 0.86*	< 0.001
Baseline FEV1 (%-predicted)	94.73 ± 14.46	95.26 ± 13.87	95.60 ± 14.28	94.18 ± 13.57	0.071
Baseline FVC (%-predicted)	96.26 ± 13.19	96.27 ± 13.30	95.81 ± 13.61	93.10 ± 12.33*†‡	< 0.001
Baseline FEV1/FVC (%)	78.68 ± 8.59	79.33 ± 7.90	79.76 ± 7.82*	80.06 ± 7.28*	< 0.001

Values are expressed as mean ± standard deviation for normally distributed continuous variables, median and interquartile range for non-normally distributed variables and percentage for categorical variables. *P*-trend was analyzed normally distributed continuous variables by the linear regression, for non-normally distributed continuous variable by Jonckheere-Terpstra tests, and for categorical variables by Cochran-Armitage test for trend

^*^, †, and ‡ meant *P* < 0.05 when compared to < 1.05, 1.06–1.45, 1.45–2.03 groups of HOMA-IR, respectively, using Bonferroni post-hoc analysis of one-way ANOVA for normally distributed continuous variables, Mann–Whitney U tests for non-normally distributed continuous variable, and Chi-square tests for categorical variables

**Fig. 3:**
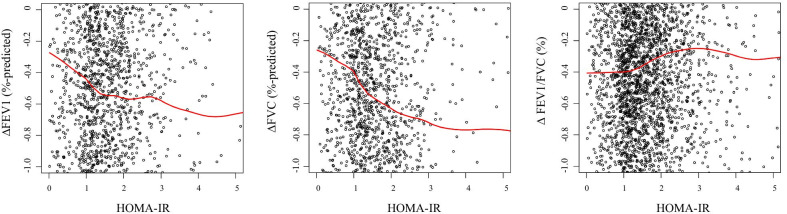
The association between HOMA-IR and annual changes in lung function. The red line represented the LOWESS regression curve. Abbreviations: HOMA-IR, homeostatic model assessment-insulin resistance; LOWESS, locally weighted scatter-plot smoothing

**Fig. 4:**
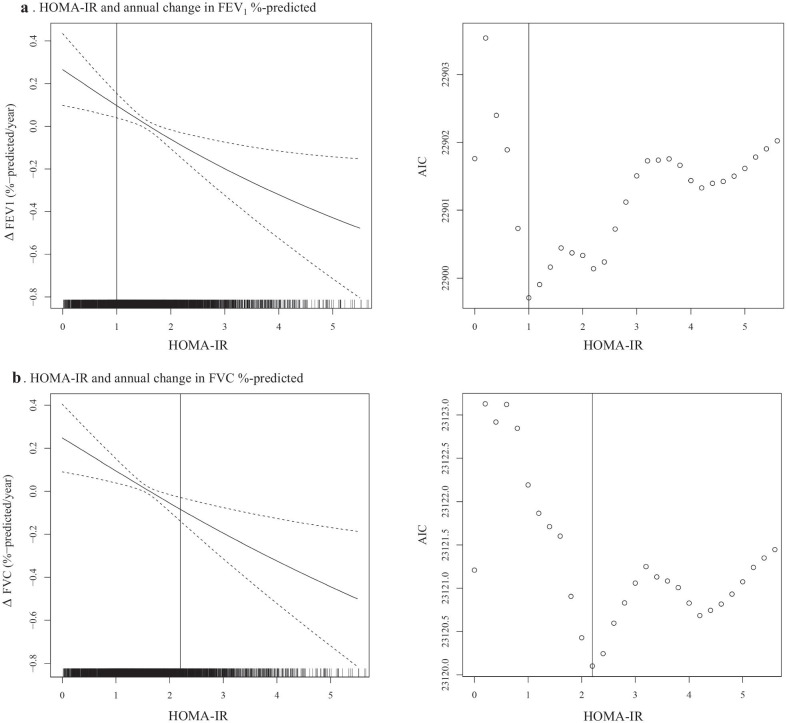
GAM plot between HOMA-IR and annual changes in lung function. The dashed lines indicate 95% CIs for values of smoothed annual changes in FEV_1_%-predicted and FVC %-predicted, using the multivariate GAM analysis after adjusting for age, sex, high income, college graduate, active physical activity, smoking status, and annual averaged values of BMI, waist circumference, systolic and diastolic BP, triglyceride, HDL cholesterol, eGFR, WBC count, hemoglobin, and CRP level. Abbreviations: GAM, general additive model; HOMA-IR, homeostatic model assessment-insulin resistance; CI, confidence interval; FEV_1_, forced expiratory volume in 1 s; FVC, forced vital capacity; BMI, body mass index; BP, blood pressure; HDL, high density lipoprotein; eGFR, estimated glomerular filtration rate; WBC, white blood cell; CRP, C-reactive protein

We explored the association between HOMA-IR and annual changes in lung function (Fig. 3). As HOMA-IR increased, annual changes in FEV_1_%-predicted and FVC %-predicted change decreased, whereas annual change in FEV_1_/FVC ratio change increased. In multivariate linear regression analysis, per one unit increased log transformed HOMA-IR was associated with decline in FEV_1_%-predicted (β: − 0.23, 95% CI: − 0.36 to − 0.11, *p* < 0.001) and FVC %-predicted (β: − 0.20, 95% CI: − 0.33 to − 0.08, *p* = 0.001) (Table 2). In the categorical analysis, the odds for the lowest quartile of annual changes in FEV_1_%-predicted (Q1: <  − 1.73%-predicted/year) and FVC %-predicted (Q1: − 1.90%-predicted/year) showed increasing trend (*P*-trend < 0.001) as quartiles of HOMA-IR were increased. The OR of HOMA-IR for lowest quartile of annual changes in FEV1%-predicted and FVC %-predicted were significantly higher in the upper quartiles compared to the lower quartile. In the multivariate GAM plot analysis, we identified that the association between HOMA-IR and annual changes in FEV_1_%-predicted and FVC %-predicted were negative linear, and the lowest AICs were found in HOMA-IR 1.0 unit for annual change in FEV_1_%-predicted and 2.2 unit for annual change in FVC %-predicted (Fig. 4).

**Table 2:** The effects of HOMA-IR on the lung function change

Exposure	Outcome: Annual lung function change			
	∆ FEV1 (%-predicted/year)		∆ FVC (%-predicted/year)	
Log transformed HOMA-IR	Beta (95% CI, P)		Beta (95% CI, P))	
Per one unit increase (n = 4827)	− 0.23 (− 0.36 to − 0.11, < 0.001)		− 0.20 (− 0.33 to − 0.08, 0.001)	

HOMA-IR quartile	Lowest quartile (Q1) of ∆ FEV1: < − 1.73%-predicted/year	*P*-trend	Lowest quartile (Q1) of ∆ FVC: < − 1.90%-predicted/year	*P*-trend
	Adjusted OR (95% CI, *P*)		Adjusted OR (95% CI, *P*)	
1Q: < 1.05 (n = 1218)	1 (reference)	< 0.001	1 (reference)	< 0.001
2Q: 1.05–1.44 (n = 1211)	1.14 (0.94–1.39, 0.191)		1.15 (0.94–1.41, 0.162)	
3Q: 1.45–2.02 (n = 1191)	1.22 (1.00–1.48, 0.055)		1.30 (1.06–1.59, 0.012)	
4Q: ≥ 2.03 (n = 1207)	1.34 (1.09–1.66, 0.006)		1.38 (1.11–1.71, 0.004)	

The rapid decline in lung function was defined in two aspects, which were the first quartile of FEV_1_%-predicted and FVC %-predicted, respectively. ORs were adjusted for age, sex, college graduate, high income, smoking status, active physical activity, and annual averaged values of BMI, systolic and diastolic BP, waist circumference, fasting glucose, HbA1c, triglyceride, HDL cholesterol, eGFR, WBC count, hemoglobin, and CRP level. *P*-trend for HOMA-IR quartile was analyzed by Cochran-Armitage test for trend

**Fig. 5:**
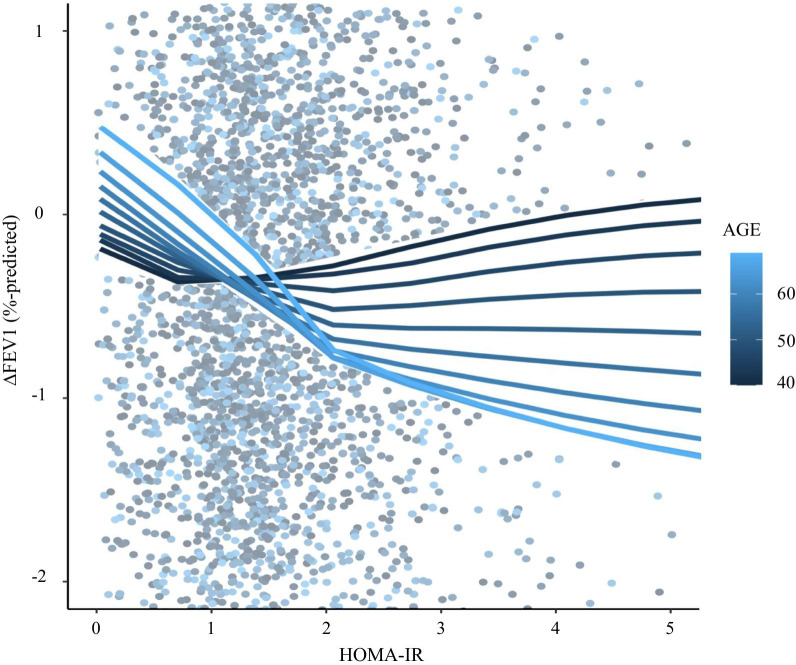
LOWESS regression plot with interaction for the effect modification of age on the association between HOMA-IR and annual change in FEV_1_%-predicted. Solid lines represented the univariate LOWESS regression curves modulated by age. The darkest blue, and lightest blue lines represented lines of mean minus one SD (44 years) and plus one SD (62 years). Abbreviations: LOWESS, locally weighted scatter-plot smoothing; HOMA-IR, homeostatic model assessment-insulin resistance; FEV1, forced expiratory volume in 1 s; SD, standard deviation

There were no subgroups showing the effect modification between HOMA-IR and annual changes in FVC % predicted (Additional file 3: Table S3). However, participant age significantly modified the effect of HOMA-IR on annual change in FEV_1_%-predicted (*p* for interaction = 0.003). Although HOMA-IR was not associated with annual change in FEV_1_%-predicted in people aged < 50 years, increased HOMA-IR was significantly associated with the decreased annual change in FEV_1_%-predicted in individuals aged ≥ 50 years (Table 3). We also identified similar effect modification of age on the association between HOMA-IR and annual change in FEV1%-predicted in the LOWESS regression plot with interaction (Fig. 5).

**Table 3:** Subgroup analysis for the effects of HOMA-IR on annual change in lung function

Exposure: per one unit increase log-transformed HOMA-IR	Adjusted beta (CI, *P*-value)	Outcome: annual change in lung function			
		∆ FEV1 (%-predicted/year)		∆ FVC (%-predicted/year)	
		*P* for interaction	Adjusted beta (CI, *P*-value)	*P* for interaction	
Subgroup	No. of people				
Age (years)	< 50 (n = 2177)	0.04 (− 0.13 to 0.22, 0.613)	0.003	0.03 (− 0.15 to 0.20, 0.775)	0.153
	≥ 50 (n = 2650)	− 0.41 (− 0.58 to − 0.24, < 0.001)		− 0.33 (− 0.51 to − 0.16, < 0.001)	
Sex	Male (n = 2186)	− 0.24 (− 0.41 to − 0.07, 0.006)	0.798	− 0.15 (− 0.33 to 0.03, 0.103)	0.554
	Female (n = 2641)	− 0.22 (− 0.40 to − 0.05, 0.012)		− 0.24 (− 0.41 to − 0.06, 0.008)	
Current or past smoker	No (n = 2812)	− 0.18 (− 0.34 to − 0.02, 0.027)	0.447	− 0.18 (− 0.34 to − 0.02, 0.012)	0.941
	Yes (n = 2015)	− 0.30 (− 0.48 to − 0.11, 0.002)		− 0.18 (− 0.37 to 0.01, 0.060)	
Active physical activity	No (n = 2512)	− 0.17 (− 0.32 to − 0.02, 0.028)	0.496	− 0.12 (− 0.28 to 0.04, 0.002)	0.423
	Yes (n = 2315)	− 0.31 (− 0.50 to − 0.11, 0.002)		− 0.29 (− 0.49 to − 0.10, 0.004)	
High BP	No (n = 4043)	− 0.21 (− 0.35 to − 0.08, 0.002)	0.579	− 0.20 (− 0.33 to − 0.06, 0.004)	0.919
	Yes (n = 784)	− 0.35 (− 0.66 to − 0.03, 0.030)		− 0.27 (− 0.60 to 0.06, 0.104)	
Annual averaged BMI (kg/m^2^)	< 24 (n = 2223)	− 0.30 (− 0.50 to − 0.11, 0.002)	0.345	− 0.22 (− 0.42 to − 0.03, 0.023)	0.998
	≥ 24 (n = 2604)	− 0.17 (− 0.33 to − 0.02, 0.031)		− 0.19 (− 0.35 to − 0.02, 0.026)	
Annual averaged eGFR (mL/min/1.73m^2^)	< 84 (n = 2496)	− 0.22 (− 0.39 to − 0.05, 0.010)	0.387	− 0.10 (− 0.27 to 0.07, 0.256)	0.751
	≥ 84 (n = 2331)	− 0.24 (− 0.42 to − 0.06, 0.010)		− 0.31 (− 0.49 to − 0.12, 0.001)	
Annual averaged WBC count (× 10^3^/μL)	< 6.3 (n = 2396)	− 0.26 (− 0.44 to − 0.08, 0.006)	0.643	− 0.15 (− 0.34 to 0.04, 0.121)	0.735
	≥ 6.3 (n = 2431)	− 0.21 (− 0.37 to − 0.05, 0.012)		− 0.22 (− 0.40 to − 0.05, 0.013)	
Annual averaged Hemoglobin (mg/dL)	< 13.5 (n = 2462)	− 0.17 (− 0.35 to 0.02, 0.080)	0.713	− 0.12 (− 0.31 to 0.07, 0.217)	0.425
	≥ 13.5 (n = 2365)	− 0.29 (− 0.45 to − 0.14, < 0.001)		− 0.24 (− 0.40 to − 0.08, 0.004)	

Adjusted beta and 95% CIs were analyzed using the multivariate linear regression. Age, sex, college graduate, high income, smoking status, active physical activity, and annual averaged values of BMI, systolic and diastolic BP, waist circumference, fasting glucose, HbA1c, triglyceride, HDL cholesterol, eGFR, WBC count, hemoglobin, and CRP level were included for adjustment. The variable used to divide subgroup was excluded from this analysis

## Discussion

Our study revealed that increased IR associated with decline in lung function using community-based prospective cohort in Korea. Recently, decreased lung function has been suggested as a complication of IR. In their large-scale cross-sectional study, Lawlor et al. found that HOMA-IR decreased by 5% and 8% for a 1 SD increase in log FEV_1_ and FVC, respectively [24]. Sagun et al. also showed that decreased FEV_1_%-predicted was correlated with increased insulin resistance (HOMA-IR ≥ 2.5) in an age-adjusted analysis (OR 0.97, 95% CI 0.94–1.00, *P* = 0.028) [25]. However, most previous studies were cross-sectional, and a causal association between insulin resistance and decreased lung function needed to be evaluated in a prospective cohort. Therefore, we conducted the current study using a prospective community-based cohort and found that increased insulin resistance was associated with a decline in lung function, particularly in the elderly population.

In the current study, increased HOMA-IR was independently associated with decline in FEV_1_%-predicted and FVC %-predicted. Cigarette smoking is a well-established risk factor for lung function deterioration. In the large scale randomized clinical trial, annual change in FEV_1_%-predicted was approximately − 1.4%-predicted/year in the continuing smoker group [26]. Compared to this result, IR can be considered a risk factor for rapid decline in lung function. In LOWESS regression analysis, increased HOMA-IR presented negative association with annual changes in FEV_1_%-predicted and FVC %-predicted, but not with FEV_1_/FVC ratio. In the natural history of chronic obstructive pulmonary disease, the rate of FEV_1_ decline is slow at high FEV_1_ and faster as FEV_1_ decreases [27]. Due to the nature of the community-based cohort, the majority of participants had a high baseline FEV_1_. These factors could explain why HOMA-IR was not associated with annual change in FEV_1_/FVC ratio.

We also found negative linear associations between HOMA-IR and annual changes in FEV_1_%-predicted and FVC %-predicted using the multivariate GAM plot, and the suggested threshold of HOMA-IR for decline in lung function was 1.0 unit for annual change in FEV_1_%-predicted and 2.2 unit for annual change in FVC %-predicted. As a surrogate marker of IR, several studies have shown that the cutoff value of HOMA-IR for the risk of developing type 2 diabetes mellitus and metabolic syndrome in the Korean population is approximately 2.5 [28–30]. Our findings showed lower threshold than those of previous studies, indicating that decline in lung function may be more sensitive to the systemic effects of IR. Moreover, these results suggest that early treatment of IR can be an effective intervention to prevent decline in lung function.

There were two possible explanations for the potential pulmonary hazard of IR. First, chronic low-grade inflammation in adipose tissue contributes to the development of IR [31, 32]. Levels of cytokines, such as tumor necrosis factor-alpha (TNF-α) and interleukin-6, are increased with IR and cause lung fibrosis and inflammation [33, 34]. Second, respiratory muscle weakness is related to IR. In high IR status, TNF-α and interferon-gamma cause muscle wasting through the degeneration of myotubes and modulation of myogenesis [35, 36].

In subgroup analysis, age showed a statistically significant effect modification on the association between HOMA-IR and annual change in FEV_1_%-predicted. Although HOMA-IR was not associated with annual change in FEV_1_%-predicted in the younger group, increased HOMA-IR was significantly related to decline in FEV_1_%-predicted in the older group.

IR is associated with chronic low-grade inflammation [31]. The risk of chronic low-grade inflammation increased with the aging process [37, 38]. Lung function can decline with increased inflammation [39, 40]. Therefore, we assumed that increased chronic low-grade inflammation during the aging process may potentiate the metabolic hazard of IR in lung function deterioration. In this regard, early intervention to reduce IR in the elderly population may have a beneficial effect on future lung function preservation.

Several limitations should be considered when interpreting our findings. First, two or fewer additional lung function measurements were performed in this study. Therefore, our study period was not sufficiently long. Further studies performing more spirometric tests will be needed to clarify the long-term effect of IR on decline in lung function. Second, our study was conducted in single ethnicity and selected areas. Therefore, the results should be elucidated cautiously in accordance with racial and regional differences.

Despite these limitations, our study has several strengths. First, to the best of our knowledge, this is the first study to investigate the association between IR and lung function using a large-scale prospective cohort based on the general population. Second, we calculated the threshold of HOMA-IR for decline in lung function using a nonlinear analytic method. Finally, the interaction between age and IR to annual change in FEV_1_%-predicted was investigated and presented using visualization.

## Conclusion

In South Korea, increased IR was independently associated with decline in FEV_1_%-predicted and FVC %-predicted. We also found that age modified the relationship between IR and FEV_1_%-predicted, particularly valid in older people. The potential pulmonary hazard of IR needs to be confirmed in future interventional studies.

## Supplementary Information

**Additional file 1**. KoGES baseline Core Questionnaire.

**Additional file 2**: **Figure S1**. Definitions of outcome and exposure variables. Abbreviations: HOMA-IR, homeostatic model assessment-insulin resistance. **Figure S2**. Correlation between age and HOMA-IR. Abbreviations: HOMA-IR, homeostatic model assessment-insulin resistance.

**Additional file 3**:** Tables S1**. Comparison of participants' baseline characteristics with or without additional spirometry and** Table S2**. Additional subgroup analysis for the effects of HOMA-IR on annual change in lung function.

## Data Availability

The data of our study is fully available when manuscript is accepted for publication.

## Abbreviations

HOMA-IR: Homeostatic model assessment-insulin resistance
FEV1: Forced expiratory volume in 1 s
FVC: Functional vital capacity
OR: Odds ratio
CI: Confidence interval
BP: Blood pressure
BMI: Body mass index
WBC: White blood cell
HbA1c: Hemoglobin A_1_c
HDL: High density lipoprotein
CRP: C-reactive protein
eGFR: Estimated glomerular filtration rate
SD: Standard deviation
IQR: Interquartile range
LOWESS: Locally weighted scatter-plot smoothing
GAM: Generalized additive model
AIC: Akaike’s information criterion
TNF-α: Tumor necrosis factor-alpha
