# Supplementary material for: Association between insulin resistance and lung function trajectory over 4 years in South Korea: community-based prospective cohort
Source: BMC Pulm Med. 2021 Apr 1;21:110. doi: 10.1186/s12890-021-01478-7 (PMC8017677; doi:10.1186/s12890-021-01478-7)
Supplement: Supplementary file 1 — Additional file 1. KoGES baseline Core Questionnaire. [file 12890_2021_1478_MOESM1_ESM.pdf]

Korean Genome and Epidemiology Study (KoGES)

Baseline Core Questionnaire

ID		Survey date: year month date			
name		sex	male / female	resident registration number	□□ □□ □□ - □□□□□□□<br>*actual D.O.B.: 19__ /__ /__ (□□years old) (solar/lunar calendar)
postal code1	-	address 1			
home phone1	( ) -	address line1			
postal code2	-	address 2			
home phone2	( ) -	address line2			
work phone	( ) -	mobile	- -		
E-mail					
Family contact / name	(Relationship:______)<br>Name:	Phone:			

※ Blue : Optional within the studies

History of disease, surgery, medication, and family

1. Have you EVER been told by a health professional you had following diseases?

① No ② Yes (If 'yes', please describe in detail)

name of disease	diagnosed or not			age at first diagnosis	treatment progress
	no	yes	don't know		
Hypertension	①	②	⑨	_____ years old	① fully recovered<br>② currently in treatment--> method of treatment?(allowing multiple responses)<br>① drug<br>② diet<br>③ exercise<br>③ had been treated before, but not now<br>④ never been treated(untreated)<br>⑨ don't know
Diabetes	①	②	⑨	_____ years old	① fully recovered<br>② currently in treatment--> method of treatment?(allowing multiple responses)<br>① drug<br>② insulin injection<br>③ diet<br>④ exercise<br>③ had been treated before, but not now<br>④ never been treated(untreated)<br>⑨ don't know
Hyperlipidemia	①	②	⑨	_____ years old	① fully recovered<br>② currently in treatment--> method of treatment?(allowing multiple responses)<br>① drug<br>② diet<br>③ exercise<br>③ had been treated before, but not now<br>④ never been treated(untreated)<br>⑨ don't know
Stroke	①	②	⑨	_____ years old	① fully recovered<br>② currently in treatment<br>③ had been treated before, but not now<br>④ never been treated(untreated)<br>⑨ don't know
Angina/myocardial infraction	①	②	⑨	_____ years old	① fully recovered<br>② currently in treatment<br>③ had been treated before, but not now<br>④ never been treated(untreated)<br>⑨ don't know
Tuberculosis	①	②	⑨	_____ years old	① fully recovered<br>② currently in treatment<br>③ had been treated before, but not now<br>④ never been treated(untreated)<br>⑨ don't know
Thyroid disease (①hyperthyroidism, ②hypothyroidism)	①	②	⑨	_____ years old	① fully recovered<br>② currently in treatment<br>③ had been treated before, but not now<br>④ never been treated(untreated)<br>⑨ don't know

name of disease	diagnosed or not			age at first diagnosis	treatment progress
	no	yes	don't know		
Chronic gastritis	①	②	⑨	______ years old	① fully recovered<br>② currently in treatment<br>③ had been treated before, but not now<br>④ never been treated(untreated)<br>⑨ don't know
Gastric ulcer	①	②	⑨	______ years old	① fully recovered<br>② currently in treatment<br>③ had been treated before, but not now<br>④ never been treated(untreated)<br>⑨ don't know
Duodenal ulcer	①	②	⑨	______ years old	① fully recovered<br>② currently in treatment<br>③ had been treated before, but not now<br>④ never been treated(untreated)<br>⑨ don't know
Colon polyps	①	②	⑨	______ years old	① fully recovered<br>② currently in treatment<br>③ had been treated before, but not now<br>④ never been treated(untreated)<br>⑨ don't know
Acute liver failure	①	②	⑨	______ years old	① fully recovered<br>② currently in treatment<br>③ had been treated before, but not now<br>④ never been treated(untreated)<br>⑨ don't know
Fatty liver disease	①	②	⑨	______ years old	① fully recovered<br>② currently in treatment<br>③ had been treated before, but not now<br>④ never been treated(untreated)<br>⑨ don't know
Chronic hepatitis/ liver cirrhosis	①	②	⑨	______ years old	① fully recovered<br>② currently in treatment<br>③ had been treated before, but not now<br>④ never been treated(untreated)<br>⑨ don't know
Gallstone disease/ cholecystitis	①	②	⑨	______ years old	① fully recovered<br>② currently in treatment<br>③ had been treated before, but not now<br>④ never been treated(untreated)<br>⑨ don't know
Chronic bronchitis	①	②	⑨	______ years old	① fully recovered<br>② currently in treatment<br>③ had been treated before, but not now<br>④ never been treated(untreated)<br>⑨ don't know
Chronic obstructive pulmonary disease (COPD)	①	②	⑨	______ years old	① fully recovered<br>② currently in treatment<br>③ had been treated before, but not now<br>④ never been treated(untreated)<br>⑨ don't know

name of disease	diagnosed or not			age at first diagnosis	treatment progress
	no	yes	don't know		
Asthma	①	②	⑨	______ years old	① fully recovered<br>② currently in treatment<br>③ had been treated before, but not now<br>④ never been treated(untreated)<br>⑨ don't know
Allergic disease (rhinitis, atopy, allergic conjunctivitis, food allergy, etc.)	①	②	⑨	______ years old	① fully recovered<br>② currently in treatment<br>③ had been treated before, but not now<br>④ never been treated(untreated)<br>⑨ don't know
Gout	①	②	⑨	______ years old	① fully recovered<br>② currently in treatment<br>③ had been treated before, but not now<br>④ never been treated(untreated)
Arthritis	①	②	⑨	______ years old	① fully recovered<br>② currently in treatment<br>③ had been treated before, but not now<br>④ never been treated(untreated)<br>⑨ don't know
Osteoporosis	①	②	⑨	______ years old	① fully recovered<br>② currently in treatment<br>③ had been treated before, but not now<br>④ never been treated(untreated)<br>⑨ don't know
Cataract	①	②	⑨	______ years old	① fully recovered<br>② currently in treatment<br>③ had been treated before, but not now<br>④ never been treated(untreated)<br>⑨ don't know
Depression	①	②	⑨	______ years old	① fully recovered<br>② currently in treatment<br>③ had been treated before, but not now<br>④ never been treated(untreated)<br>⑨ don't know
Periodontal (gum) disease	①	②	⑨	______ years old	① fully recovered<br>② currently in treatment<br>③ had been treated before, but not now<br>④ never been treated(untreated)<br>⑨ don't know
Malignant tumor (1) __________cancer	①	②	⑨	______ years old	① fully recovered<br>② currently in treatment<br>③ had been treated before, but not now<br>④ never been treated(untreated)<br>⑨ don't know
Malignant tumor (2) __________cancer	①	②	⑨	______ years old	① fully recovered<br>② currently in treatment<br>③ had been treated before, but not now<br>④ never been treated(untreated)<br>⑨ don't know

types of malignant tumors:
(1) stomach (2) liver (3) colorectal (4) breast (5) cervical
(6) lung (7) thyroid (8) prostate (9) bladder (10) others ____________

location of fracture	diagnosed or not			age at first diagnosis	treatment progress
	no	yes	don't know		
Fracture (1)<br>location___________	①	②	⑨	______ years old	① fully recovered<br>② currently in treatment<br>③ had been treated before, but not now<br>④ never been treated(untreated)<br>⑨ don't know
					cause: ①accident ②fall or sprain ⑨don't know
Fracture (2)<br>location___________	①	②	⑨	______ years old	① fully recovered<br>② currently in treatment<br>③ had been treated before, but not now<br>④ never been treated(untreated)<br>⑨ don't know
					cause: ①accident ②fall or sprain ⑨don't know

area of fracture:
(1) wrist (2) shoulder area (3) femoral (pelvis) (4) vertebral compression (5) others ________

2. Have you had any SURGERY or other surgical procedures?
① No ② Yes (If 'yes', please describe in detail)

Name of surgery (operation)
(1) cholelithiasis (2) angina (3) partial hepatectomy (4) gastrectomy
(5) cholecystectomy (6) prostate surgery (7) thyroid surgery (8) mastectomy
(9) phimosiectomy (10) vasectomy (11) tubal surgery (12) blood transfusion
(13) acupuncture (14) hepaitis B vaccination (15) others ___________

Name of surgery (operation)	Age at first operation
operation 1_____	_____ years old
operation 2_____	_____ years old
operation 3_____	_____ years old
operation 4_____	_____ years old
operation 5_____	_____ years old

3. Have you **taken continuously (3 months or more)** the following drugs?
① no ② yes (If 'yes', please describe in detail) ↴

NIH Korea National Institute of Health

Type	name of drug	total frequency of taking						dose /time	total period of taking
		none	1-3 times /wk	4-6 times /wk	1 time /day	2 times /day	more than 3 times /day		
Aspirin		⓪	①	②	③	④	⑤		__years __months
Pain reliever<br>(Name: __________)		⓪	①	②	③	④	⑤		__years __months

4. **In the past 12 months**, have you been **regularly** taking the following **dietary supplements**?
① no ② yes (If 'yes', please describe in detail) ↴

Type	total frequency of taking						dose /time	total period of taking
	none	1-3 times /wk	4-6 times /wk	1 time /day	2 times /day	more than 3 times /day		
multivitamin<br>(aronamin gold, centrum, etc.)	⓪	①	②	③	④	⑤		__years __months
vitamin C	⓪	①	②	③	④	⑤		__years __months
vitamin E (tocopherol, etc.)	⓪	①	②	③	④	⑤		__years __months
calcium supplement (oscal, etc.)	⓪	①	②	③	④	⑤		__years __months
iron supplement	⓪	①	②	③	④	⑤		__years __months
glucosamine	⓪	①	②	③	④	⑤		__years __months
ginseng product	⓪	①	②	③	④	⑤		__years __months
others ___________	⓪	①	②	③	④	⑤		__years __months

5. Has anyone of your blood relatives including father, mother, sisters, brothers, or childer ever been told by a health professional that they had the following diseases? or has anyone of your family members died from the following diseases?
① no ② yes (If 'yes', please describe in detail)↴

name of disease	diagnosed or not	number	Relationship*			age at first diagnosis
hypertension	① no<br>② yes<br>⑨ don't know	A	① father<br>④ sister	② mother<br>⑤ son	③ brother<br>⑥ daughter	_____ years old
		B	① father<br>④ sister	② mother<br>⑤ son	③ brother<br>⑥ daughter	_____ years old
		C	① father<br>④ sister	② mother<br>⑤ son	③ brother<br>⑥ daughter	_____ years old
diabetes	① no	A	① father	② mother	③ brother	_____ years old

	② yes ⑨ don't know		④ sister ⑤ son ⑥ daughter	
		B	① father ② mother ③ brother ④ sister ⑤ son ⑥ daughter	_____ years old
		C	① father ② mother ③ brother ④ sister ⑤ son ⑥ daughter	_____ years old
angina/ myocardial infraction	① no ② yes ⑨ don't know	A	① father ② mother ③ brother ④ sister ⑤ son ⑥ daughter	_____ years old
		B	① father ② mother ③ brother ④ sister ⑤ son ⑥ daughter	_____ years old
		C	① father ② mother ③ brother ④ sister ⑤ son ⑥ daughter	_____ years old
stroke	① no ② yes ⑨ don't know	A	① father ② mother ③ brother ④ sister ⑤ son ⑥ daughter	_____ years old
		B	① father ② mother ③ brother ④ sister ⑤ son ⑥ daughter	_____ years old
		C	① father ② mother ③ brother ④ sister ⑤ son ⑥ daughter	_____ years old
hyperlipidem ia	① no ② yes ⑨ don't know	A	① father ② mother ③ brother ④ sister ⑤ son ⑥ daughter	_____ years old
		B	① father ② mother ③ brother ④ sister ⑤ son ⑥ daughter	_____ years old
		C	① father ② mother ③ brother ④ sister ⑤ son ⑥ daughter	_____ years old
osteoporosis	① no ② yes ⑨ don't know	A	① father ② mother ③ brother ④ sister ⑤ son ⑥ daughter	_____ years old
		B	① father ② mother ③ brother ④ sister ⑤ son ⑥ daughter	_____ years old
		C	① father ② mother ③ brother ④ sister ⑤ son ⑥ daughter	_____ years old
cancer	① no ② yes ⑨ don't know	A ____cancer	① father ② mother ③ brother ④ sister ⑤ son ⑥ daughter	_____ years old
		B ____cancer	① father ② mother ③ brother ④ sister ⑤ son ⑥ daughter	_____ years old
		C ____cancer	① father ② mother ③ brother ④ sister ⑤ son ⑥ daughter	_____ years old
other disease1 (_________)	① no ② yes ⑨ don't know	A	① father ② mother ③ brother ④ sister ⑤ son ⑥ daughter	_____ years old
		B	① father ② mother ③ brother ④ sister ⑤ son ⑥ daughter	_____ years old
		C	① father ② mother ③ brother ④ sister ⑤ son ⑥ daughter	_____ years old
other disease2 (_________)	① no ② yes ⑨ don't know	A	① father ② mother ③ brother ④ sister ⑤ son ⑥ daughter	_____ years old
		B	① father ② mother ③ brother ④ sister ⑤ son ⑥ daughter	_____ years old
		C	① father ② mother ③ brother ④ sister ⑤ son ⑥ daughter	_____ years old

types of malignant tumors:
(1) stomach (2) liver (3) colorectal (4) breast (5) cervical
(6) lung (7) thyroid (8) prostate (9) bladder (10) others _____________

5-1. Including yourself, how many siblings do you have? What is your birth order among your siblings? ______ brothers ______ sisters ________ th

5-2. How many children have you got?____ sons _____ daughters

6. Has your father ever been diagnosed with femoral fracture?
① no ② yes

7. Has your mother ever been diagnosed with femoral fracture?
① no ② yes

General Information

8. What is your highest level of education?
⓪ never attended school ① left elementary school ② graduated elementary or left middle school
③ graduated middle or left high school ④ graduated high shool ⑤ graduated technical school
⑥ left university ⑦ bachelor's degree ⑧ higher than bachelor's degree

9. What is **your father's** highest level of education?
⓪ never attended school ① left elementary school ② graduated elementary or left middle school
③ graduated middle or left high school ④ graduated high shool ⑤ graduated technical school
⑥ left university ⑦ bachelor's degree ⑧ higher than bachelor's degree

10. What is **your mother's** highest level of education?
⓪ never attended school ① left elementary school ② graduated elementary or left middle school
③ graduated middle or left high school ④ graduated high shool ⑤ graduated technical school
⑥ left university ⑦ bachelor's degree ⑧ higher than bachelor's degree

11. What is your current **marital status**?
① never married ② married ③ separated ④ divorced
⑤ widowed ⑥ living with partner ⑦others(___________)

11-1 How old were you when you (first) got married? _______ years old

12. What is your monthly household income?
① less than ₩500,000 ② ₩500,000 - less than 1,000,000
③ ₩1,000,000 - less than 1,500,000 ④ ₩1,500,000 - less than 2,000,000
⑤ ₩2,000,000 - less than 3,000,000 ⑥ ₩3,000,000 - less than 4,000,000
⑦ ₩4,000,000 - less than 6,000,000 ⑧ more than ₩6,000,000

13. Including yourself, how many people live in your household?
_________people, including myself

14. How long have you been lived at your current address? Since year of ________

15. Where have you lived the longest? How long did you live there?

___________ Province ___________ city/town From year of ________ to _______

Job History

16. Currently, what kind of job do you do? (classification code: please refer to the following job classifications)

classification code	industry	department (performed task)	age at first started
□□			_____ years old

① *parliament member, executive and high official (congressman, administrative and management, general management, etc.)*
② *expert (science, computer, engineering, health and medical, education, administrative/management and finance, law/social service and religious, cultural/art and broadcasting, etc.)*
③ *technician and semiprofessional (science, computer, engineering, health and medical, education, management and financial, social service and religious, art/entertainment and sports game, etc.)*
④ *office worker (general office work, customer service, etc.)*
⑤ *service worker (interactive service, cooking and food service, travel and transportation, security service, etc.)*
⑥ *sales worker (wholesale, retail, telecommunication, model and advertising, insurance and real estate, etc.)*
⑦ *skilled laborer in agriculture, forestry and fishery (agriculture, forestry, fishery, etc.)*
⑧ *technician (extraction and construction, metal, machine, machine installation and maintenance, precision instrument/craftsmanship and handicraft, etc.)*
⑨ *mechanic (handling + assembling) (machine and system operation, assembling, etc.)*
⑩ *labor service (agriculture, forestry, fishery, manufacturing, mining/construction and shipping, etc.)*
⑪ *military service*
⑫ *housewife or labor in housekeeping*
⑬ *unemployed*
⑭ *others (   )*

17. What kind of job have you worked the longest? (classification code: please refer to the previous job classifications)

classification code	industry	department (performed task)	period
□□			from___to ___ years old

NIH Korea National Institute of Health

Weight History

18. Currently, how much do you weigh and tall are you?

weight : ______ kg, height : _______ cm,

How much did you weigh 6 months ago? ______ kg,

How much did you weigh 2 years ago? ______ kg,

How much did you weigh at age of 35? ______ kg,

How much did you weigh at age of 18 (age around your high school graduation)? ______ kg,

If you are older than 50, how much did you weigh at age of 50? ______ kg, ⓪ N/A

19. When did you weigh the most and the least since age of 18? (For female: exclude weight during pregnancy)

highest weight : age ______, _________kg,
lowest weight : age ______, _________kg,

Lifestyle (smoking, drinking, etc.)

20. Have you smoked more than more than 5packs (100 cigarettes) of cigarettes in your entire life?
① No (☞ go to question 22)

□ Yes →

- Do you currently smoke cigarettes?

② No (quitted □□years □□months ago) ③ Yes

20-1. Approximately, how many cigarettes have you smoked in your entire life?
① 100~400 cigarettes ② more than 400 cigarettes

20-2. How old were you when you first started to smoke cigarettes fairly regularly?
□□ years old

20-3. How long has it been since you smoked cigarettes?
total □□years □□months

20-4. On the average, how many cigarettes did you usually smoke per day?
approximately □□□ cigarettes

21. If you currently smoke cigarettes, please answer the following questions

☐ Yes →

21-1. How soon after you wake up do you smoke?
① within 5 minutes (3pts) ② from 6 to 30 minutes (2pts)
③ from more than 30 minutes to 1 hour (1pt) ④ more than 1 hour (0pt)

21-2. Is it hard for you to refrain from smoking in restricted places such as church, theater, library, etc?
① yes (1pt) ② no (0pt)

21-3. Which case of smoking makes you not want to quit the smoking the most?
① first cigarette in the morning (1pt) ② others (0pt)

21-4. How many cigarettes do you usually smoke per day?
① less than 10 cigarettes (0pt) ② 11-20 cigarettes (1pt)
③ 21-30 cigarettes (2pts) ④ more than 30 cigarettes (3pts)

21-5. Do you smoke more in the first few hours after waking up in the morning than other times during the day?
① yes (1pt) ② no (0pt)

21-6. Do you smoke when you are sick in bed all day?
① yes (1pt) ② no (0pt)

22. Are you currently exposed to secondhand smoking?
① No (☞ go to question 23)
② Yes

22-1. How often are you exposed to indoor secondhand smoke (such as home or office)?
⓪ none ① 1-2 times/wk ② 3-4 times/wk ③ 5-6 times/wk ④ everyday

22-2. How long are you exposed to indoor secondhand smoke during the day?
☐☐☐☐ minutes

22-3. How long have you been exposed to secondhand smoking? ____ years ____ months

23. Did any adults smoke cigarettes at home during your childhood?
① yes ② no

24. Since age of 20, have you lived or are you living with a regular smoker?
① yes ② no

25. Can you not drink from the beginning or do not drink at all?
※ Exclude few glasses from communions or ancestral rites.
① No, I do not drink from the beginning or do not drink at all (☞ go to question 28)
○ I have drunk before

- Do you still drink? ② no (quitted ☐☐years ☐☐months before) ③ yes ⑨

25-1. Until now, how many years have you been drinking ? ☐☐☐years

25-2. Please check your average frequency and amount per occasion of each type of drinks in the past year.

Type of drinks	average frequency in the past 12 months								total amount per occasion
	none	1 time /month	2-3 times /month	1 time /week	2-3 times /week	4-6 times /week	1 time /day	more than 2 times /day	
soju (Korean liquor)	⓪	①	②	③	④	⑤	⑥	⑦	______glasses (50 cc)
beer	⓪	①	②	③	④	⑤	⑥	⑦	______glasses (200 cc)
rice wine	⓪	①	②	③	④	⑤	⑥	⑦	______glasses (250 cc)
sake	⓪	①	②	③	④	⑤	⑥	⑦	______glasses (50 cc)
wine	⓪	①	②	③	④	⑤	⑥	⑦	______glasses (90 cc)
hard liquor	⓪	①	②	③	④	⑤	⑥	⑦	______glasses (30 cc)
fruit wine	⓪	①	②	③	④	⑤	⑥	⑦	______glasses (50 cc)
other (　　　)	⓪	①	②	③	④	⑤	⑥	⑦	other ____glasses (____glasses)

* type of glasses for other ; ① rice wine ② soju ③ wine ④ beer ⑤ hard liquor ⑨ N/A

* 1bottle rice wine(1,800 cc) = 7glasses, 1bottle wine(750 cc) = 8glasses, 1bottles soju(350 cc) = 7glasses, 1bottle beer(500 cc) = 2.5glasses, 1bottle hard liquor(700 cc) = 23glasses

26. Does your face turn red instantly after consuming small amount of drink as a cup of beer?
① no ② yes

27. Do you get intoxicated easily and become cheerful after couple of drinks?
① no ② yes

Lifestyle (Sleep & Physical Activity)

28. **In the past 12 months**, how many hours of sleep (including nap) do you get in a 24-hour period on average? ☐☐hours ☐☐minutes

29. In the past 12 months, when have you usually gone to bed at night and gotten up in the morning?
gone to bed at night: hour: ☐☐ minute: ☐☐
gotten up in the morning: hour: ☐☐ minute: ☐☐

30. Currently, do you have trouble falling asleep?
⓪ no ① occasionally (1-2 days/week)
② often (3-4 days/week) ③ almost everyday (more than 5 days/week)

31. Do you have trouble falling back to sleep after waking up during night?
⓪ no ① occasionally (1-2days/week)
② often (3-4 days/week) ③ almost everyday (more than 5 days/week)

32. Has anyone told you that you snore loudly?
① no (☞ go to question 33) ② yes

32-1. How often do you snore?
① rarely ② 1-3 times/month ③ 1-3 times/week
④ 4-5 times/week ⑤ almost everyday (more than 6 times a week)

32-2. Has anyone sharing a room with you told you that you stopped breathing while snoring?
① no ② yes

32-3. Have you been waken up during your sleep due to snoring?
① no ② yes

33. How fast do you think you usually walk indoors?
⓪ not able to walk ① slow (slower than 3 km/h)
② normal (faster than 3 km/h, slower than 4 km/h)
③ fast (faster than 4 km/h, slower than 5 km/h) ④ very fast (faster than 5 km/h)

34. How would you rate your regular physical activities?
① no activities (hospitalized patient and not able to move)
② less active (spend most time in stationary activities such as sitting down, regular office workers who do not actively carry out regular exercise during leisure time)
③ active (mostly spend time sitting down but also doing task while standing up, commute, shopping, household chores, regular light activities)
④ very active (work standing up or actively spend leisure time)
⑨

35. Do you regularly exercise enough to sweat?
① No (☞ go to question 36)
② Yes

35-1. If you do, how many time a week do you exercise?
① 1-2 times/week ② 3-4 times/week ③ 5-6 times/week ④ almost everyday

35-2. Average time per exercise: □□□□ minutes

35-3. What kind of exercise do you perform?

Type	times/week	total time period	time per exercise
(#1 :)	(times/wk)	(years months)	(hours minutes)
(#2 :)	(times/wk)	(years months)	(hours minutes)
(#3 :)	(times/wk)	(years months)	(hours minutes)
(#4 :)	(times/wk)	(years months)	(hours minutes)
(#5 :)	(times/wk)	(years months)	(hours minutes)
(#6 :)	(times/wk)	(years months)	(hours minutes)
(#7 :)	(times/wk)	(years months)	(hours minutes)

※ Type of exercises

(1) aerobics (2) dancing (3) jogging (running) (4) fast walking
(5) taking a walk (6) hiking (7) swimming (8) fitness
(9) workout(with equipments) (10) free gymnastics, stretching (11) bike riding (12) Judo, Taekwondo
(13) yoga (14) jumping rope (15) sit-up (16) push-up
(17) lifting dumbbell (18) tennis (19) golf (field) (20) golf (indoor)
(21) bowling (22) soccer (23) table tennis (24) badminton
(25) baseball (26) basketball (27) skiing (28) fishing
(29) skating (30) inline skating (31) other (________)

36. In the past year, what was your average time per week spent in each of the following household chores? (multiple answers allowed)
① hand-washing clothes __times/week __minutes/time
② house cleaning __times/week __minutes/time
③ kitchen chores __times/week __minutes/time
④ gardening __times/week __minutes/time
○9

37. In the past 12 months, how many stairs did you climb up and down on average per day?
( ) stairs

38. In the past 12 months, how often did you take a walk?
① I did not take a walk
② __times/week _____ minutes

Social psychological stress (PWI-SF)

39. Over the last 4 weeks, have you felt that it was mentally or physically difficult to manage?
① no ② occasionally ③ often

40. In general, would you say your current health status is?
① very healthy ② healthy ③ normal
④ unhealthy ⑤ very unhealthy

41. In general, would you say your current financial status is?
① affluent ② average ③ challenging ④ very challenging

42. Circle the number for each statement which best describes how often you felt or experienced about physical and psychological state in recent few weeks (PWI-SF)

list	Always	Frequently	Sometimes	Never	
1. I feel very comfortable and healthy now	⓪	①	②	③	⑨
2. I do not feel refreshed after waking up	⓪	①	②	③	⑨
3. I am very tired and exhausted so that it's even hard to eat	⓪	①	②	③	⑨
4. I cannot sleep well due to worries	⓪	①	②	③	⑨
5. I feel my mind is clear and conscious	⓪	①	②	③	⑨
6. I feel full of energy	⓪	①	②	③	⑨
7. I feel uneasy and anxious at night	⓪	①	②	③	⑨
8. I feel I manage myself quite well as most people do	⓪	①	②	③	⑨
9. I feel that things are generally going well for me	⓪	①	②	③	⑨
10. I am satisfied with the methods or procedures I carried out	⓪	①	②	③	⑨
11. I can begin doing something right away	⓪	①	②	③	⑨
12. I can enjoy normal day to day life	⓪	①	②	③	⑨
13. I become anxious or grumpy	⓪	①	②	③	⑨
14. I can solve problems thrown at me	⓪	①	②	③	⑨
15. I feel depressed and unhappy	⓪	①	②	③	⑨
16. I am losing faith in myself	⓪	①	②	③	⑨
17. All things considered, I feel happy	⓪	①	②	③	⑨
18. I feel life is worth living	⓪	①	②	③	⑨

NIH Korea National Institute of Health

43. Circle the number for each statement which best describes how often you felt or behaved this way DURING THE PAST WEEK <<CES-D-K>>

list	less than 1 day/wk	1-2days/wk	3-4days/wk	more than 5days/wk	
1. I was bothered by things that usually don't bother me	①	②	③	④	⑨
2. I did not feel like eating; my appetite was poor	①	②	③	④	⑨
3. I felt that I could not shake off the blues, even with help from my family and friends	①	②	③	④	⑨
4. I had trouble keeping my mind on what I was doing	①	②	③	④	⑨
5. I have been relatively well	①	②	③	④	⑨
6. I felt depressed	①	②	③	④	⑨
7. Everything felt challenging	①	②	③	④	⑨
8. I felt hopeless about the future	①	②	③	④	⑨
9. I thought my life had been a failure	①	②	③	④	⑨
10. I felt that I was just as good as other people	①	②	③	④	⑨
11. I had rough night sleeping (could not sleep well)	①	②	③	④	⑨
12. I felt fearful	①	②	③	④	⑨
13. I talked less than usual	①	②	③	④	⑨
14. I felt lonely	①	②	③	④	⑨
15. I had no big complaint	①	②	③	④	⑨
16. People were unfriendly	①	②	③	④	⑨
17. I started crying all of sudden	①	②	③	④	⑨
18. I felt sad	①	②	③	④	⑨
19. I felt that people disliked me	①	②	③	④	⑨
20. I could not "get going"	①	②	③	④	⑨
total					

Women's Health

1. How old were you when you had your first menstrual period? □□ years old

2. How old were you when you started to have menstrual period regularly? □□ years old

3. Have you ever been pregnant? ① no (☞ go to question 16)
② yes (☞ go to question 4)

4. How old were you at the time of your first pregnancy? □□ years old

4-1. What was the result of your first pregnancy?
① childbirth ② stillbirth (baby born without signs of life) ③ ectopic pregnancy
④ currently in first pregnancy ⑤ miscarriage ⑥ abortion

5. During pregnancy, have you ever been told by a doctor that you had gestational diabetes?
① no ② yes

6. During pregnancy, have you ever been told by a doctor you had **preeclampsia**?
① no ② yes

7. How many children have you given birth to? ⓪ none (☞ go to question 16)
① given birth to (□□children)

8. How old were you at the time of your first and last live birth?
first live birth: □□ years old
last live birth: □□ years old

9. Have you ever had a cesarean deliveries? ① no
② yes (□□times)

10. Have you had a **macrosomic baby** (birth weight more than 4 kg)?
① no
② yes (______th children, at age of______)

11. In case of a full term delivery (more than 36 weeks), have you had a **low birth weight baby** (birth weight less than 2.5 kg)?
① no
② yes (______th children, at age of______)

12. Have you ever breastfed your children? ① no
② yes

12-1. If yes, how many children have you breastfed? total □□ children

12-2. How old were you when you started breastfeeding? □□ years old

12-3. How many months did you breastfeed when you first did it? □□□ months

12-4. If you add up all the periods you breastfed each child, how many months is it?
total □□□ months

calculation example	first child 3 months<br>second child 6 months → total 9 months<br>third child 0 month

13. Have you ever had a miscarriage? ① no
② yes (total □□ times)

※ **miscarriage:** termination of pregnancy before 20 weeks

13-1. If yes, how old were you at the time of your first miscarriage ?
_______years old

14. Have you ever had an abortion (induced abortion, dilation & curettage)? ① no
② yes (total □□ times)

14-1. If yes, how old were you at the time of your first abortion ?
_______years old

15. Have you ever had a stillbirth? ① no
② yes (total □□ times)

※ **stillbirth:** delivering baby without signs of life after 20 weeks of pregnancy

15-1. If yes, how old were you at the time of your first stillbirth?
_______years old

16. Have you ever taken oral contraceptive pills?
① no (☞ go to question 17)
○ yes → Do you still take it? ② No, I do not take it anymore.
③ Yes, I currently take it.

16-1. How old were you when you started to take? □□ years old

16-2. Total dosage period of oral contraceptives? total □□ years □□ months □□ days

calculation example	age 25-28 ; 3 years<br>33-35 ; 2 years → total 7 years<br>40-present(42) ; 2 years<br>* If you don't remember accurately, please write 99.

17. Have you ever had a hysterectomy that is, surgery to remove your uterus or womb?
① no
② yes (□□ years old)

18. Have you ever had your ovaries removed?
   ① no
   ② yes, one ovary removed
   ③ yes, both sides, partially removed
   ④ yes, both sides, completely removed (□□ years old)

19. Have you ever been told by a doctor you had a benign breast tumor?
   ① no
   ② yes, □□ years old

20. Have you had menstrual periods in the past 3 months?
   ① **no** (I did not have periods in the past 12 months or more)
   ② **no**, I had a few periods in the past 12 months, but none in the past 3 months
   ③ **yes,** I had irregular periods in the past 12 months
   ④ **yes,** I always had periods regularly ⇒ average menstrual cycle: □□□ days (ex: 30 days)
   ⑨

20-1. How old were you when you had your last menstrual period? (□□ years old)

20-2. What is the reason that you have not had a period?
   ① getting old ② operation
   ③ radiotherapy ④ pharmacotheraphy

20-3. Have you ever used female hormones or injections since the menopause?
   ① no
   ② had used in the past (for □□□ months)
   ③ currently using (for □□□ months)
