# Supplementary figures and images for: Association between insulin resistance and lung function trajectory over 4 years in South Korea: community-based prospective cohort
Source: BMC Pulm Med. 2021 Apr 1;21:110. doi: 10.1186/s12890-021-01478-7 (PMC8017677; doi:10.1186/s12890-021-01478-7)

Figure S1.

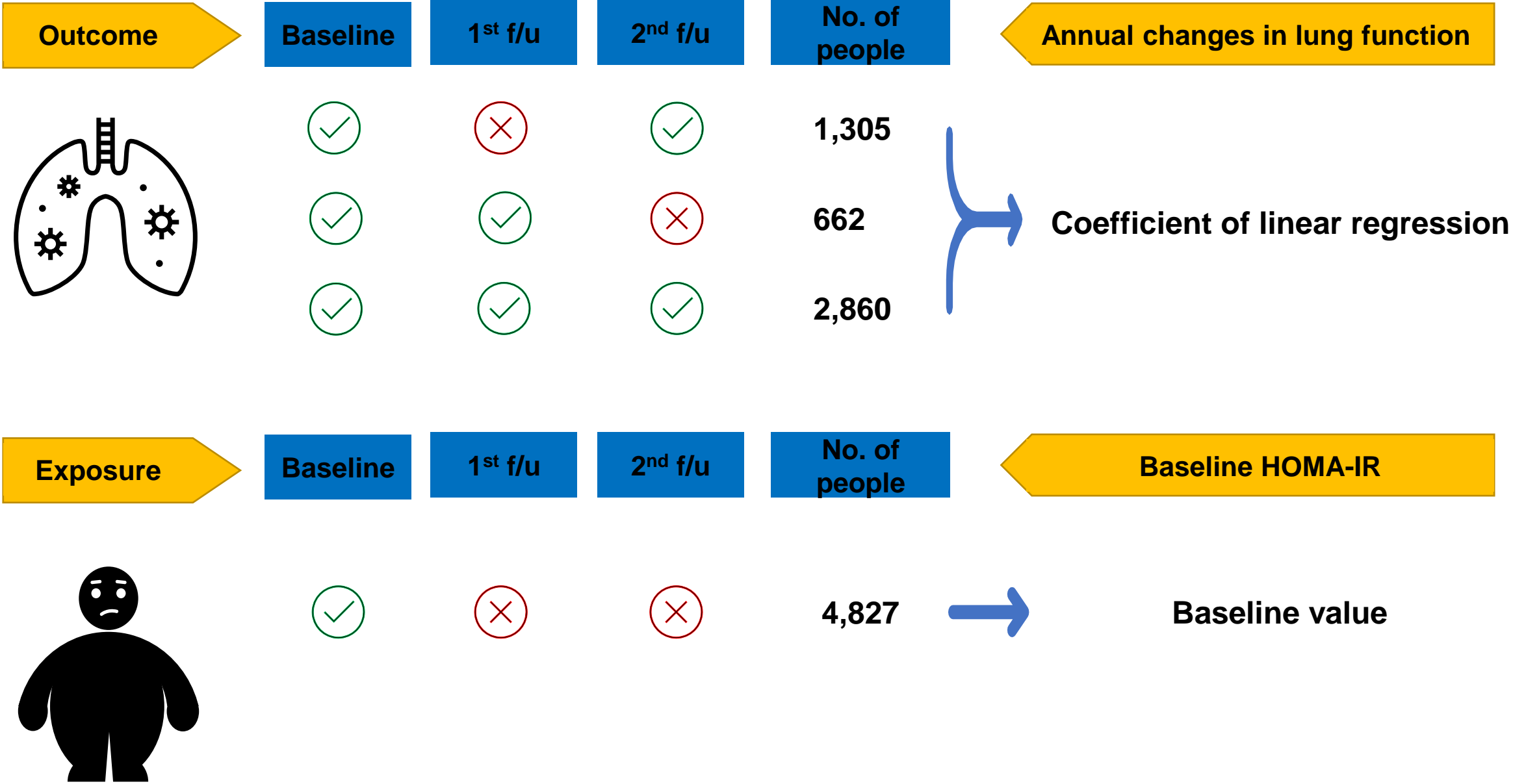

Figure S2.

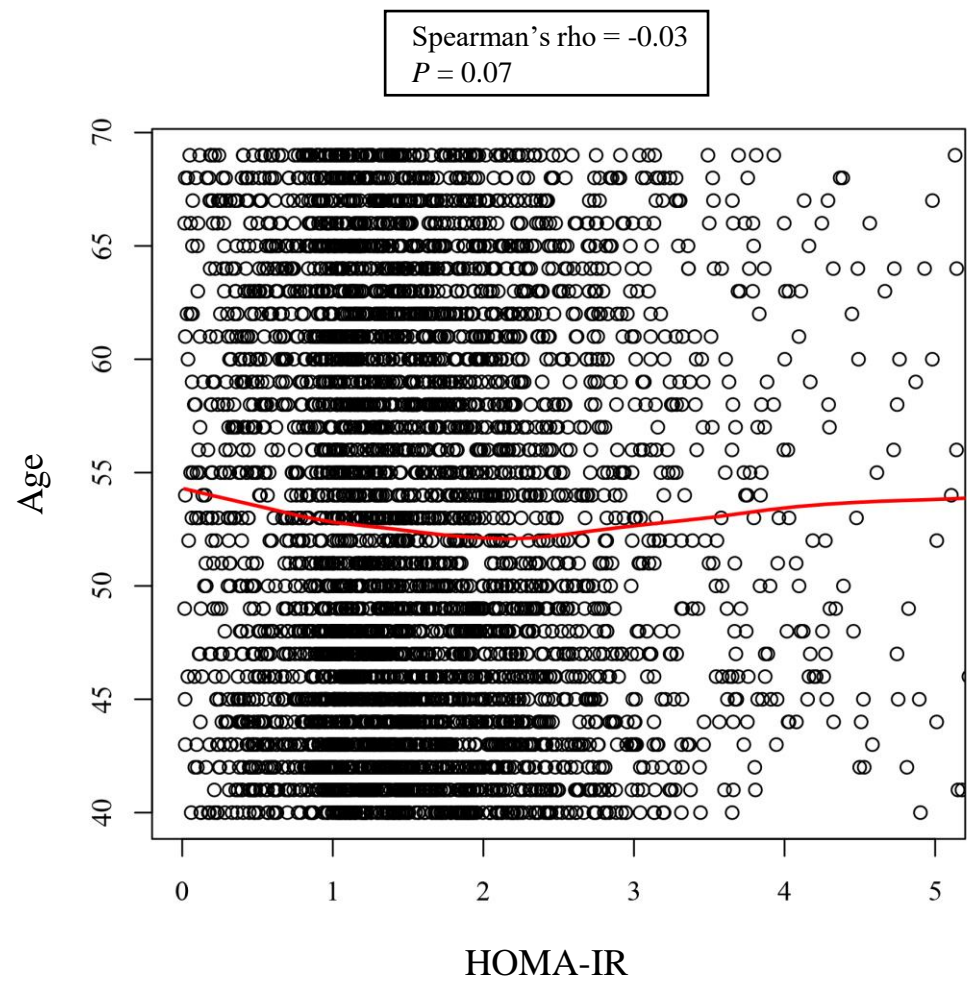

Supplement: Supplementary file 2 — Additional file 2: Figure S1. Definitions of outcome and exposure variables. Abbreviations: HOMA-IR, homeostatic model assessment-insulin resistance. Figure S2. Correlation between age and HOMA-IR. Abbreviations: HOMA-IR, homeostatic model assessment-insulin resistance. [file 12890_2021_1478_MOESM2_ESM.pdf]
