# Supplementary material for: Association between insulin resistance and lung function trajectory over 4 years in South Korea: community-based prospective cohort
Source: BMC Pulm Med. 2021 Apr 1;21:110. doi: 10.1186/s12890-021-01478-7 (PMC8017677; doi:10.1186/s12890-021-01478-7)
Supplement: Supplementary file 3 — Additional file 3: Tables S1. Comparison of participants' baseline characteristics with or without additional spirometry and Table S2. Additional subgroup analysis for the effects of HOMA-IR on annual change in lung function. [file 12890_2021_1478_MOESM3_ESM.pdf]

**Supplementary**

**Table S1. Comparison of participants' baseline characteristics with or without additional spirometry**

| | With additional spirometry (n = 4,827) | Without additional spirometry (n = 1,609) | *P*-value |
|---|---|---|---|
| Age (years) | 52.65 ± 8.94 | 52.91 ± 9.32 | 0.330 |
| Male, n (%) | 2186 (45.3) | 725 (45.1) | 0.897 |
| High income, n (%) | 673 (13.9) | 238 (14.8) | 0.421 |
| College graduate, n (%) | 494 (10.2) | 159 (9.9) | 0.721 |
| Current or past smoker, n (%) | 2015 (41.7) | 686 (42.6) | 0.550 |
| Active physical activity, n (%) | 2315 (48.0) | 764 (47.5) | 0.762 |
| Baseline BMI ($Kg/m^2$) | 24.44 ± 3.12 | 24.37 ± 3.28 | 0.496 |
| Baseline systolic BP (mmHg) | 122.53 ± 18.90 | 122.98 ± 19.51 | 0.405 |
| Baseline diastolic BP (mmHg) | 80.59 ± 11.49 | 80.67 ± 11.85 | 0.808 |
| Baseline waist circumference (cm) | 82.43 ± 8.79 | 82.05 ± 9.10 | 0.135 |
| Baseline fasting glucose (mg/dL) | 82 (77-90) | 82 (77-91) | 0.437 |
| Baseline HbA1c (%) | 5.6 (5.4-5.9) | 5.6 (5.4-5.9) | 0.730 |
| Baseline triglyceride (mg/dL) | 138 (101-193) | 134 (101-187) | 0.256 |
| Baseline HDL cholesterol (mg/dL) | 44.49 ± 9.87 | 45.32 ± 10.79 | 0.006 |
| Baseline eGFR ($mL/min/1.73m^2$) | 92.01 ± 14.15 | 91.76 ± 14.96 | 0.557 |
| Baseline hemoglobin (g/dL) | 13.56 ± 1.57 | 13.50 ± 1.58 | 0.207 |
| Baseline WBC count ($\times 10^3/\mu L$) | 6.74 ± 1.88 | 6.78 ± 1.94 | 0.495 |
| Baseline CRP level (mg/dL) | 0.14 (0.07-0.26) | 0.14 (0.07-0.27) | 0.409 |
| Baseline FEV1 (%-predicted) | 94.94 ± 14.06 | 94.21 ± 15.82 | 0.102 |
| Baseline FVC (%-predicted) | 95.36 ± 13.18 | 94.82 ± 14.24 | 0.177 |
| Baseline FEV1/FVC (%) | 79.46 ± 7.93 | 79.22 ± 9.41 | 0.366 |

In the comparison, statistics were calculated excluding missing values for each variable. Values are expressed as mean ± standard deviation for normally distributed continuous variables, median and interquartile range for non-normally distributed variables and percentage for categorical variables. *P*-value was analyzed normally distributed continuous variables by student's t-test, for non-normally distributed continuous variable by Wilcoxon rank sum test, and for categorical variables by Pearson's Chi-square test.

**Table S2. Additional subgroup analysis for the effects of HOMA-IR on annual change in lung function.**

| | | Outcome: annual change in lung function | | | |
|---|---|---|---|---|---|
| | | Δ FEV1 (%-predicted/year) | | Δ FVC (%-predicted/year) | |
| Exposure: per one unit increase log-transformed HOMA-IR | | Adjusted beta (CI, *P*-value) | *P* for interaction | Adjusted beta (CI, *P*-value) | *P* for interaction |
| Subgroup | No. of people | | | | |
| High income | No (n = 4,154) | -0.26 (-0.39 to -0.12, < 0.001) | 0.151 | -0.20 (-0.34 to -0.07, 0.004) | 0.955 |
| | Yes (n = 673) | -0.05 (-0.32 to 0.21, 0.690) | | -0.22 (-0.49 to 0.05, 0.115) | |
| College graduate | No (n = 4,333) | -0.24 (-0.37 to -0.11, < 0.001) | 0.861 | -0.23 (-0.38 to -0.09, 0.003) | 0.874 |
| | Yes (n = 494) | -0.23 (-0.58 to 0.13, 0.212) | | -0.17 (-0.52 to 0.18, 0.336) | |
| Annual averaged Waist circumference (cm) | < 83 (n = 2,374) | -0.20 (-0.37 to -0.02, 0.025) | 0.398 | -0.16 (-0.34 to 0.01, 0.066) | 0.398 |
| | ≥ 83 (n = 2,453) | -0.29 (-0.47 to -0.12, < 0.001) | | -0.31 (-0.49 to -0.13, < 0.001) | |
| Annual averaged fasting glucose (mg/dL) | < 88 (n = 2,430) | -0.16 (-0.32 to 0.01, 0.066) | 0.984 | -0.16 (-0.33 to 0.01, 0.060) | 0.256 |
| | ≥ 88 (n = 2,397) | -0.29 (-0.47 to -0.11, 0.002) | | -0.26 (-0.44 to -0.07, 0.006) | |
| Annual averaged triglyceride (mg/dL) | < 130 (n = 2,402) | -0.22 (-0.41 to -0.03, 0.024) | 0.847 | -0.17 (-0.37 to 0.02, 0.075) | 0.849 |
| | ≥ 130 (n = 2,425) | -0.23 (-0.39 to -0.08, 0.004) | | -0.22 (-0.39 to -0.06, 0.008) | |
| Annual averaged HDL cholesterol (mg/dL) | < 44 (n = 2,453) | -0.27 (-0.45 to -0.10, 0.002) | 0.948 | -0.23 (-0.41 to -0.06, 0.009) | 0.892 |
| | ≥ 44 (n = 2,374) | -0.18 (-0.36 to -0.01, 0.042) | | -0.16 (-0.34 to 0.02, 0.082) | |
| Annual averaged CRP (mg/dL) | < 0.1 (n = 2,070) | -0.33 (-0.52 to -0.15, < 0.001) | 0.522 | -0.31 (-0.50 to -0.13, 0.001) | 0.410 |
| | ≥ 0.1 (n = 2,757) | -0.13 (-0.29 to 0.03, 0.118) | | -0.09 (-0.26 to 0.08, 0.295) | |

Adjusted beta and 95% CIs were analyzed using the multivariate linear regression. Age, sex, college graduate, high income, smoking status, active physical activity and annual averaged values of BMI, systolic and diastolic BP, waist circumference, fasting glucose, HbA1c, triglyceride, HDL cholesterol, eGFR, WBC count, hemoglobin, and CRP level were included for adjustment. The variable used to divide subgroup was excluded from this analysis.
